# Voxel-wise quantification of myocardial blood flow with cardiovascular magnetic resonance: effect of variations in methodology and validation with positron emission tomography

**DOI:** 10.1186/1532-429X-16-11

**Published:** 2014-01-24

**Authors:** Christopher A Miller, Josephine H Naish, Mark P Ainslie, Christine Tonge, Deborah Tout, Parthiban Arumugam, Anita Banerji, Robin M Egdell, David Clark, Peter Weale, Christopher D Steadman, Gerry P McCann, Simon G Ray, Geoffrey JM Parker, Matthias Schmitt

**Affiliations:** 1 North West Heart Centre, University Hospital of South Manchester, Wythenshawe Hospital, Manchester, UK; 2 Centre for Imaging Sciences & Biomedical Imaging Institute, University of Manchester, Manchester, UK; 3 Institute of Cardiovascular Sciences, University of Manchester, Manchester, UK; 4 Nuclear Medicine Centre, Central Manchester University Hospitals, Manchester, UK; 5 East Cheshire NHS Trust, Macclesfield, UK; 6 Alliance Medical Cardiac MRI Unit, Wythenshawe Hospital, Manchester, UK; 7 Siemens Healthcare, Camberlely, Surrey, UK; 8 NIHR Leicester Cardiovascular Biomedical Research Unit and Department of Cardiovascular Sciences, University of Leicester, Leicester, UK

**Keywords:** Cardiovascular magnetic resonance, Coronary artery disease, Myocardial blood flow, Positron emission tomography, Quantification

## Abstract

**Background:**

Quantitative assessment of myocardial blood flow (MBF) from cardiovascular magnetic resonance (CMR) perfusion images appears to offer advantages over qualitative assessment. Currently however, clinical translation is lacking, at least in part due to considerable disparity in quantification methodology. The aim of this study was to evaluate the effect of common methodological differences in CMR voxel-wise measurement of MBF, using position emission tomography (PET) as external validation.

**Methods:**

Eighteen subjects, including 9 with significant coronary artery disease (CAD) and 9 healthy volunteers prospectively underwent perfusion CMR. Comparison was made between MBF quantified using: 1. Calculated contrast agent concentration curves (to correct for signal saturation) versus raw signal intensity curves; 2. Mid-ventricular versus basal-ventricular short-axis arterial input function (AIF) extraction; 3. Three different deconvolution approaches; Fermi function parameterization, truncated singular value decomposition (TSVD) and first-order Tikhonov regularization with b-splines. CAD patients also prospectively underwent rubidium-82 PET (median interval 7 days).

**Results:**

MBF was significantly higher when calculated using signal intensity compared to contrast agent concentration curves, and when the AIF was extracted from mid- compared to basal-ventricular images. MBF did not differ significantly between Fermi and Tikhonov, or between Fermi and TVSD deconvolution methods although there was a small difference between TSVD and Tikhonov (0.06 mL/min/g). Agreement between all deconvolution methods was high. MBF derived using each CMR deconvolution method showed a significant linear relationship (p < 0.001) with PET-derived MBF however each method underestimated MBF compared to PET (by 0.19 to 0.35 mL/min/g).

**Conclusions:**

Variations in more complex methodological factors such as deconvolution method have no greater effect on estimated MBF than simple factors such as AIF location and observer variability. Standardization of the quantification process will aid comparison between studies and may help CMR MBF quantification enter clinical use.

## Background

Myocardial ischaemia is a fundamental determinant of prognosis and non-invasive imaging assessment of ischaemia is integral to the management of patients with suspected or established coronary artery disease (CAD) [1]. Cardiovascular magnetic resonance (CMR) perfusion imaging, involving qualitative assessment of the first-pass of contrast agent though the myocardium, has emerged as an effective method of diagnosing CAD [2]. Visual interpretation however is limited to providing information on the regional distribution of MBF only, a limitation particularly relevant to conditions where blood flow is diffusely abnormal e.g. widespread CAD or microvascular coronary dysfunction. Quantitative assessment of myocardial blood flow (MBF) overcomes this limitation, indeed it has been used to provide pathophysiological insight into conditions where microvascular disease is manifest [3]. In addition, quantitative measurement of MBF appears to allow more accurate evaluation of ischaemic burden [4], is potentially advantageous in patients with differing degrees of epicardial stenoses and may also allow more precise characterization of changes in MBF following therapeutic interventions. Studies using positron emission tomography (PET) have demonstrated the superiority of MBF quantification over qualitative and semi-quantitative methods for identification of CAD [5,6].

CMR MBF quantification has been validated in animal models against microspheres and in healthy volunteers and patients with CAD against PET, on per segment or per sector levels [7-9]. More recently voxel-wise CMR assessment of MBF has been validated against microspheres in canines, although to date voxel-wise assessment has not been validated in humans with CAD [10]. Voxel-wise assessment potentially allows superior identification of the extent of ischaemia and as such may be particularly helpful in conditions where ischaemia is confined to limited regions of myocardium e.g. the subendocardium, although at the potential cost of reduced signal to noise. However, despite quantitative perfusion CMR being applied increasingly widely in the research setting, it has not yet become a clinical tool. One of the main reasons for this is the considerable disparity that exists in quantification methodology.

The aim of this study was primarily to evaluate the effect that common methodological differences in CMR MBF quantification have on voxel-wise measurement of MBF in patients with CAD and in healthy volunteers. Specifically we aimed to assess the impact of accounting for the non-linear relationship between contrast agent concentration and signal intensity, arterial input function (AIF) location and method of deconvolution. Interobserver variability was assessed in order to put the magnitude of the effect of these methodological variations into context. Finally, CMR-derived MBF was compared with MBF quantified using PET.

## Methods

### Patients and study design

Patients with CAD with typical symptoms of angina and with known significant stenoses (>75% luminal narrowing) of one or more epicardial coronary arteries, as determined angiographically (qualitative analysis), were prospectively recruited. All patients awaiting elective percutaneous coronary intervention (PCI) at a tertiary UK cardiac centre (University Hospital of South Manchester NHS Trust) over a 6-month period were screened for study eligibility. Exclusion criteria included left main stem disease, severe proximal 3-vessel disease, acute coronary syndrome within 6-weeks, estimated glomerular filtration rate of 35 mL/min/1.73 m^2^ or less and contraindications to CMR or adenosine infusion.

In addition, healthy volunteers were recruited through hospital and university advertisement. Volunteers were completely asymptomatic with no known risk factors or history of cardiac disease, normal physical examination and normal ECG (i.e. they were not *patients* who had been referred for CMR that was subsequently found to be normal).

Patients underwent CMR and rubidium-82 (Rb-82) PET, the order of which was determined randomly. No patient had an interim cardiovascular event or coronary revascularization procedure. Healthy volunteers underwent CMR only. An ethics committee of the UK National Research Ethics Service approved the study (11/NW/0045) and written informed consent was obtained from all participants. The work was conducted according to the Declaration of Helsinki.

### Cardiovascular magnetic resonance image acquisition

Subjects were instructed to abstain from caffeine for a minimum of 12 hours prior to CMR and PET imaging. CMR was performed using a 1.5 T scanner (Avanto; Siemens Healthcare, Germany) equipped with a 32-element phased-array coil. Using a saturation recovery gradient echo sequence, basal, mid and apical left ventricular (LV) short-axis images were acquired every heartbeat during pharmacological vasodilation (‘stress’) and at rest. For stress imaging, intravenous adenosine (140 μg/kg/min) was administered via a large peripheral vein for 3-minutes prior to, and during, data acquisition. A 0.05 mmol/kg bolus of gadolinium-based contrast agent (gadopentetate dimeglumine; Gd-DTPA; Magnevist; Bayer Healthcare, Germany) was administered intravenously at 5 mL/s followed by a 30 mL saline flush. Rest imaging was performed 10 minutes after stress imaging with a further 0.05 mmol/kg of contrast agent. Typical image parameters included: FOV 270 × 360 mm, matrix 120 × 160, slice thickness 10 mm, acquired image resolution 2.5 × 2.5 × 10 mm, saturation recovery time 120 ms, echo time 1.07 ms, repetition time 2.13 ms, flip angle 12°, parallel imaging factor 2 with 24 reference lines. Following rest perfusion image acquisition, a further 0.1 mmol/kg of contrast agent was administered (‘top-up’) to bring the total dose to 0.2 mmol/kg.

In addition, steady-state free precession (SSFP) cine images were acquired in standard long-axis views and in a stack of short-axis slices covering the LV. Standard late gadolinium enhancement (LGE) imaging was performed at least 10 minutes following the contrast agent ‘top-up’ using spoiled gradient echo segmented inversion recovery, and phase sensitive inversion recovery (PSIR) segmented gradient echo, sequences.

### Positron emission tomography image acquisition

Rb-82 was supplied from a CardioGen-82® strontium-82/Rb-82 generator manufactured for Bracco (Bracco Diagnostics Inc, USA). Imaging was performed using a Siemens Biograph mCT scanner (Siemens Healthcare, Germany) with Lutetium Oxyorthosilicate crystals and extended axial FOV. Patients underwent serial rest then stress imaging as per the routine clinical protocol at our institution. A computed tomography (CT) scout view over the chest was performed for positioning followed by a low-dose CT scan (120 kV, quality reference effective mAs = 11, rotation 0.5 s, pitch 1.5, collimation 16.0 × 1.2 mm) to provide attenuation-correction of the rest emission data. 1110 MBq Rb-82 was infused intravenously at a flow rate of 50 mL/min. List mode 3D data acquisition was started with the tracer infusion and continued for 7 min. For stress imaging, intravenous adenosine (140 μg/kg/min) was administered via a large peripheral vein for 4.5 min. Intravenous Rb-82 infusion and list mode acquisition began 3 min after the start of the adenosine infusion following the same protocol as rest imaging. Registration between PET and CT images was checked for evidence of patient motion and manual adjustments were made prior to reconstruction to correct for minor motion. In cases of significant patient motion between PET and CT, an additional low dose CT was acquired at the end of the study. Both rest and stress dynamic images used for MBF quantification were reconstructed into 19 time frames (1 × 10 s, 10 × 5 s, 3 × 20 s, 2 × 30 s, 3 × 60 s) on a 128×128 matrix using ordered subset expectation maximization (OSEM) reconstruction (2 iterations, 24 subsets) with 3mm Gaussian post-filter.

### Data analysis

#### CMR myocardial blood flow quantification

Endocardial and epicardial contours were drawn on the perfusion images using Osirix Imaging Software (Pixmeo; Switzerland; v4.0). Additional regions of interest (ROI) were drawn in the blood pool on the basal and mid-ventricular images, avoiding papillary muscles and trabeculae, for AIF determination. The anterior right ventricular septal insertion point was marked. ROIs were manually translated on each perfusion image of the same slice in order to compensate for rigid-body translational motion.

### Calculation of contrast agent concentration

Saturation of the signal occurs at high contrast agent concentrations due to the non-linear relationship between contrast agent concentration and signal intensity. If not accounted for this leads to an underestimate of the AIF peak and a resulting overestimate of MBF. The method described by Biglands et al. [11] was used to convert the signal intensity in both the blood pool and myocardium to contrast agent concentration in order to account for the non-linearity and correct for signal saturation. An assumed value for native blood T_1_ was used to calculate the sequence calibration constant (dependent on receiver gain, proton density and flip angle) from the pre-contrast signal intensity in the blood pool of the stress image dataset and to convert the stress signal AIF to contrast agent concentration using a value for the relaxivity of gadopentetate dimeglumine of 4.5 s^-1^mM^-1^[12]. The calibration constant was assumed constant across both myocardium and blood and between the stress and rest image acquisitions in order to convert the remaining signal curves to contrast agent concentration.

### Deconvolution methods

Model-independent analysis of dynamic contrast enhanced CMR data is based on the central volume principle, which relates the amount of tracer in a tissue region over time to the arterial input of tracer to the region [13]. For a single input system which is *linear* (i.e. the response scales with the input) and *stationary* (i.e. the response is independent of time of arrival), the tissue contrast agent concentration curve, C(t) can be expressed as the convolution of the arterial input function, AIF(t), and the tissue impulse response function. The initial value of the tissue impulse response function is equal to the blood flow into the region, MBF such that

(1) $$C\left(t\right)=\;\mathrm{MBF}\left(\mathrm{AIF}\left(t\right)⊗R\left(t\right)\right)$$

where R(t) is the normalized impulse response function. R(t) represents the probability that a tracer molecule that entered the tissue region at t = 0 is still present in the tissue at time t and is also known as the tissue residue function. MBF can therefore be determined by a direct deconvolution of the measured contrast agent concentration in the LV cavity, i.e. the AIF(t), from the measured myocardial tissue contrast agent concentration, C(t). Deconvolution is, however, numerically unstable and requires some form of regularization. In this study three commonly used approaches were applied and compared.

### Fermi function parameterization

This approach assumes a parametric form for the impulse response function and is based on the observation by Axel [14] that the shape of the expected tissue impulse response resembles a function from quantum mechanics known as a Fermi function

(2) $$R\left(t\right)=\frac{1}{e^{\left(t-t_{0}\right)}/{}^{\tau }+1}$$

where t_0_ and *τ* determine the shape of R(t) but have no direct physiological relevance. MBF may then be determined by a non-linear least squares fit to the myocardial tissue concentration curve where the fitting function is formed using Eq. (1) with R(t) given by Eq. (2).

### Truncated singular valued decomposition

Direct deconvolution of the AIF(t) from the tissue concentration curve is achieved using a singular valued decomposition (SVD) to solve the least squares minimization problem

(3) $$\min_{X}∥\mathrm{AX}‒B∥$$

where the matrix A is the convolution matrix formed from the AIF, the vector B is given by C(t) and the vector X is the impulse response function. The simplest method of regularization is the truncated singular valued decomposition (TSVD), most commonly applied in quantification of cerebral blood flow in which all the singular values below a particular threshold are set to zero (truncated) [15,16]. The threshold value is usually set to be a fraction of the largest singular value [15,17].

### Tikhonov regularization with b-splines

Tikhonov regularization is an alternative to TSVD in which a quadratic constraint is added to Eq. (3)

(4) $$\min_{X}\left\{∥\mathrm{AX}‒B{∥}^{2}-\lambda^{2}∥\mathrm{LX}{∥}^{2}\right\}$$

resulting in a smooth truncation of the singular values with a regularization parameter, *λ*. In zeroth-order Tikhonov regularization L is the identity matrix and in first order Tikhonov regularization L is the finite difference operator. The inclusion of the finite difference operator, which is an approximation to a first derivative, favours solutions that are relatively flat. One advantage of Tikhonov regularization is the fact that, unlike TSVD, the solution depends on the choice of the regularization parameter in a continuous manner, facilitating a selection of an optimum value for the regularization parameter using L-curve analysis [18]. As a further constraint, the impulse response function was parameterized as a sum of 15 b-splines. This approach, introduced by Jerosch-Herold et al. [19], imposes smoothness and continuity on the impulse response function and has been applied in a number of quantitative CMR perfusion studies [20].

Each of the three deconvolution approaches (Fermi function fitting, TSVD and first-order Tikhonov regularization with b-splines) was applied on a voxel-wise basis within the myocardial ROIs on each slice to generate maps of MBF. Data was restricted to the first pass of the contrast agent through the heart, which was automatically detected using the AIF from the LV blood pool. All analysis was carried out using algorithms written in-house using Matlab (The Mathworks; USA; v2009A). Tikhonov regularization utilized Matlab routines from the “Regularization Tools” library by Hansen [21].

MBF maps were segmented according to the American Heart Association/American College of Cardiology 16-segment model and median voxel MBF in each segment was recorded. Comparison was made between segmental MBF calculated using: 1. contrast agent concentration curves versus signal intensity curves; 2. mid-ventricular versus basal-ventricular AIF extraction; 3. each deconvolution method. Except for when the effect of saturation correction and AIF location respectively were specifically being assessed, saturation correction and basal slice AIF extraction were used throughout.

#### PET myocardial blood flow quantification

MBF quantification was performed using a commercially available software package (Syngo MBF, Siemens Healthcare, Germany), which has been validated using N-13 ammonia PET and shown to have high observer repeatability [22,23]. The software describes the pharmacokinetic behaviour of Rb-82 using a single compartment model

(5) $$C_{T}\left(t\right)=\mathrm{Ca}\left(t\right)⊗{K_{1e}}^{-\mathrm{kt}}$$

where C_T_(t) is the myocardial activity concentration and Ca(t) is the arterial blood concentration. The response function of the myocardium is modelled by an exponential and K_1_ is the uptake ratio from blood into the tissue [22]. Processing was highly automated although operator intervention was possible to confirm or modify the automatic re-orientation of the LV and to apply motion correction if needed. The myocardium was defined automatically and sampled into 505 segments according to a cylindrical-spherical model. Myocardial tissue time-activity curves were obtained at each time frame. The arterial input function was obtained from the dynamic sequence by averaging the activity in a 1 × 3 cm cylindrical region-of-interest placed automatically in the basal LV cavity. Kinetic model fitting using non-linear regression was performed on each of the 505 polar-map sectors to compute MBF values for each voxel, which were then averaged to calculate segmental values. The software incorporates spill-over and partial volume correction [23].

Segmental MBF and myocardial perfusion reserve (MPR, calculated by dividing stress MBF by rest MBF) measured using CMR and PET were compared.

### LV volumetric analysis

LV mass, end-diastolic volume (EDV), end-systolic volume (ESV) and ejection fraction (EF) were quantified from CMR SSFP images using CMRtools (Cardiovascular Imaging Solutions, UK).

### Interobserver variability

All CMR studies were independently analysed by a second observer in order to assess Interobserver repeatability.

### Statistical analysis

All data was analysed in a blinded fashion, with independent analysis of CMR and PET data. Statistical analysis was performed using SPSS (IBM, USA; v19). Continuous variables are expressed as mean ± SD unless stated. An independent-samples t test (or Mann–Whitney U test where appropriate) was used to compare patient and volunteer demographic data. Agreement was evaluated using Bland-Altman analysis by calculating mean difference (bias) and 95% limits of agreement (i.e. mean difference ± 2 SD). The significance of the differences was assessed using generalized estimating equations (GEE) in order to adjust for the repeated measurements within each subject. For the same reason, regression analysis using GEE was used to assess the relationship between CMR-derived MBF and PET-derived MBF. Within-subject and between-subject correlations were calculated using the methods described by Bland et al. [24,25].

## Results

### Study population

Eighteen subjects were recruited, comprising 9 patients with CAD and 9 healthy volunteers. Participant characteristics are displayed in Table 1.

**Table 1:** Characteristics of participants

	**CAD patients**	**Healthy volunteers**	**p value**
	**n = 9**	**n = 9**	
Male	8 (89%)	7 (78%)	0.527
Age	68 ± 5	48 ± 9	<0.001
BMI (kg/m^2^)	26.6 ± 2.7	27.0 ± 3.4	0.783
eGFR (mL/min/m^2^)	78 ± 22	86 ± 13	0.375
Hypertension	5 (56%)	0	
Diabetes	2 (22%)	0	
Current/previous smoker	1 (11%) / 4 (44%)	0	
Previous MI	4 (44%)	0	
Previous PCI	4 (44%)	0	
Previous CABG	1 (11%)	0	
Coronary disease			
Left anterior descending	4 (44%)	-	
Circumflex	2 (22%)	-	
Right	6 (67%)	-	
Angina (CCS)			
Class 1	1 (11%)	-	
Class 2	4 (44%)	-	
Class 3	4 (44%)	-	
LVEDVI (mL/m^2^)	87 ± 19	86 ± 8	0.871
LVESVI (mL/m^2^)	36 ± 19	28 ± 4	0.223
LVEF (%)	61 ± 11	68 ± 4	0.086
LV Mass I (g/m^2^)	46 ± 9	48 ± 8	0.694

BMI indicates body mass index; eGFR estimated glomerular filtration rate; MI myocardial infarction; PCI percutaneous coronary intervention; CABG coronary artery bypass graft; CCS Canadian Cardiovascular Society; EDV end diastolic volume; ESV end-systolic volume. The suffix I indicates indexed to body surface area.

### Contrast agent concentration versus signal intensity

**Figure 1:**
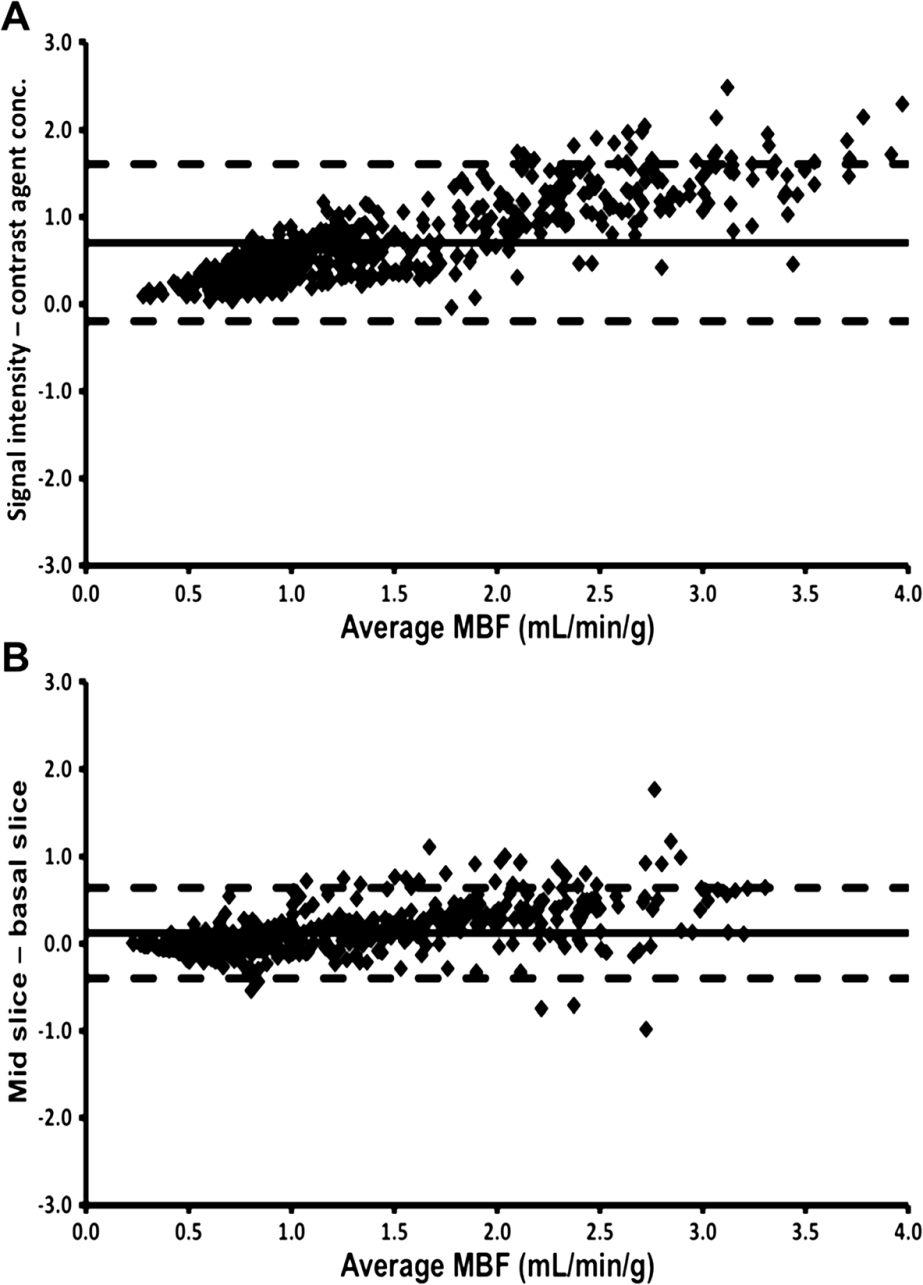
**Saturation effects and AIF location.** Bland-Altman plots displaying the agreement between MBF quantified using raw signal intensity curves versus calculated contrast agent concentration curves (to correct for signal saturation) **(A)**, and using AIF extracted from mid-ventricular short-axis images compared to basal-ventricular images **(B)**. Solid line represents mean difference; dashed lines represent ± 2 standard deviations.

Mean MBF calculated using contrast agent concentration curves was significantly higher than when signal intensity curves were used (Table 2). The degree of overestimation increased as MBF increased (Figure 1A).

**Table 2:** Effect of saturation correction and arterial input function location on myocardial blood flow quantification

	**Overall MBF (mL/min/g)**	**Stress MBF (mL/min/g)**	**Rest MBF (mL/min/g)**
**A. Saturation correction**			
Contrast agent concentration	1.10 ± 0.64	1.55 ± 0.63	0.66 ± 0.20
Signal intensity	1.80 ± 1.03	2.51 ± 1.00	1.09 ± 0.33
p value	<0.001	<0.001	<0.001
95% limits of agreement	−0.20 to 1.60	0.01 to 1.92	0.02 to 0.85
**B. AIF location**			
Basal ventricular	1.10 ± 0.64	1.55 ± 0.63	0.66 ± 0.20
Mid ventricular	1.22 ± 0.80	1.79 ± 0.75	0.64 ± 0.23
p value	0.002	<0.001	0.392
95% limits of agreement	−0.40 to 0.64	−0.36 to 0.85	−0.23 to 0.21

95% limits of agreement represent mean difference ± 2 standard deviations. For part A, the limits of agreement refer to MBF measured using raw signal intensity curves minus MBF measured using calculated contrast agent concentration curves (to correct for signal saturation); and for part B, MBF measured using a mid-ventricular-positioned AIF minus MBF measured using a basal-ventricular-positioned AIF.

### AIF location

Mean MBF was significantly higher when the AIF was extracted from the blood pool in the mid-ventricular short-axis images compared to when it was extracted from the blood pool in the basal-ventricular images (Table 2). The magnitude of the difference increased as MBF increased, indeed at rest there was no significant difference (Figure 1B).

### Method of deconvolution

**Figure 2:**
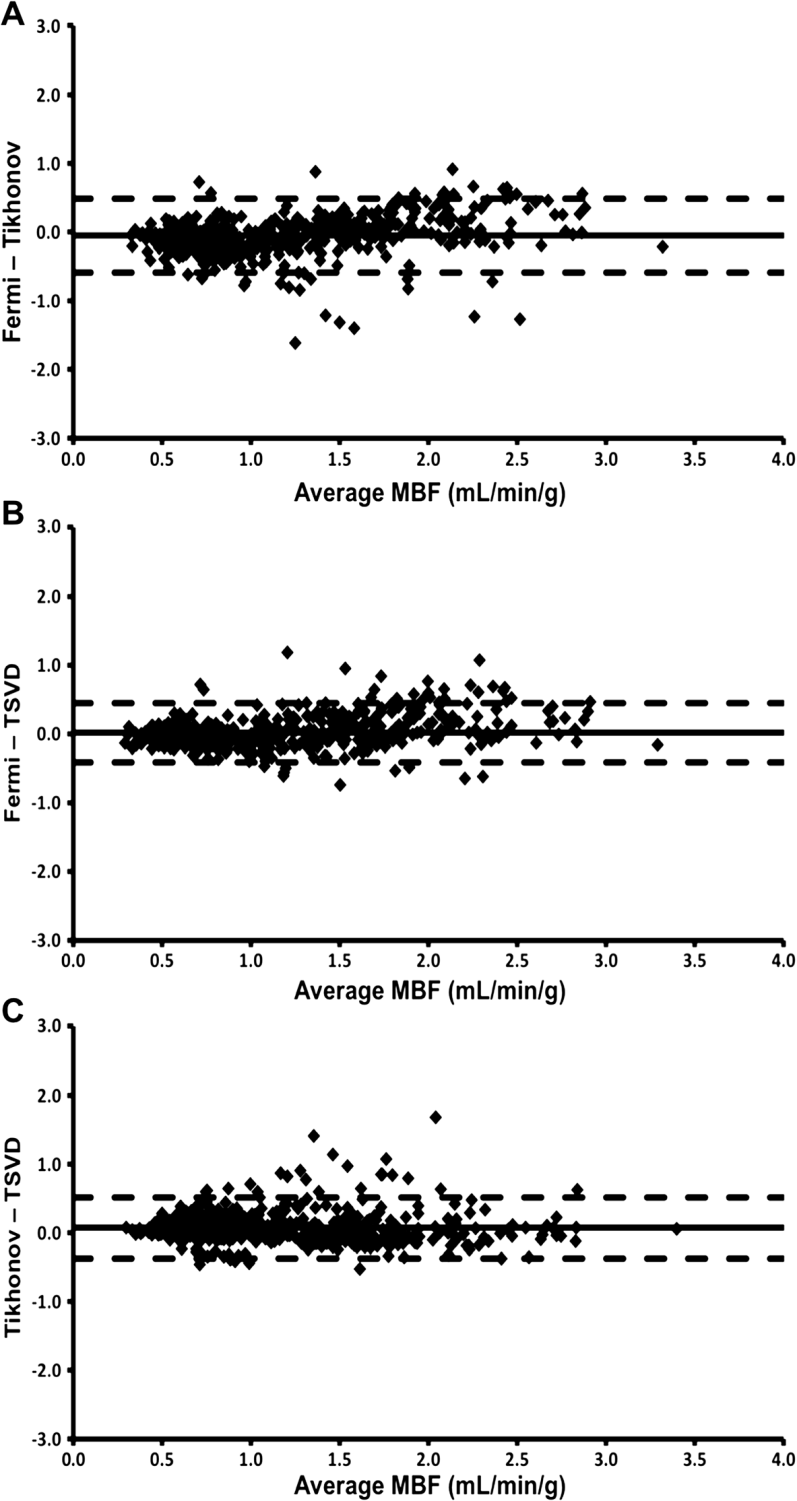
**Comparison of deconvolution methods.** Bland-Altman plots displaying the agreement between MBF measured using Fermi function (Fermi), Tikhonov regularization (Tikhonov) and TSVD methods of deconvolution. Solid line represents mean difference; dashed lines represent ± 2 standard deviations.

Overall mean MBF did not differ significantly between Fermi and Tikhonov methods and between Fermi and TSVD methods (Table 3). Overall mean MBF calculated using TSVD was significantly lower than MBF calculated using Tikhonov regularization, although the absolute difference was small (0.06 mL/min/g). Limits of agreement between all deconvolution methods were narrow (Figure 2).

**Table 3:** Effect of method of deconvolution on myocardial blood flow quantification

**A. Mean values**				
	**Fermi**	**Tikhonov**	**TSVD**	**p value**
Overall MBF (mL/min/g)	1.10 ± 0.64	1.15 ± 0.57	1.09 ± 0.56	0.023
Stress MBF (mL/min/g)	1.55 ± 0.63	1.55 ± 0.53	1.48 ± 0.54	0.105
Rest MBF (mL/min/g)	0.66 ± 0.20	0.76 ± 0.24	0.70 ± 0.20	<0.001
**B. Mean differences and limits of agreement**				
	**Mean difference**	**p-value**	**95% limits of agreement**	
**Overall MBF (mL/min/g)**				
Fermi – Tikhonov	−0.05	0.160	−0.58 to 0.48	
Fermi – TSVD	0.01	1.000	−0.41 to 0.44	
Tikhonov – TSVD	0.06	0.025	−0.37 to 0.51	
**Stress MBF (mL/min/g)**				
Fermi – Tikhonov	0.00	1.00	−0.62 to 0.61	
Fermi – TSVD	0.07	0.345	−0.44 to 0.59	
Tikhonov – TSVD	0.08	0.148	−0.42 to 0.57	
**Rest MBF (mL/min/g)**				
Fermi – Tikhonov	−0.10	<0.001	−0.52 to 0.32	
Fermi – TSVD	−0.04	0.021	−0.32 to 0.24	
Tikhonov – TSVD	0.06	0.119	−0.32 to 0.45	

95% limits of agreement represent mean difference ± 2 standard deviations. Other abbreviations as per Tables 1 and 2. p values in Part A refer to the significance of the mean difference between the three deconvolution methods; p values in part B are those obtained on post hoc analysis.

Mean MBF during stress did not differ significantly between deconvolution methods but at rest MBF calculated using the Fermi technique was significantly higher than with the other methods, although the absolute differences were again small. Limits of agreement between all methods were narrow for both stress and rest MBF values.

### Interobserver repeatability

Observer agreement for measurement of MBF was moderate, with 95% limits of agreement (± 2 standard deviations) between observers for overall MBF as follows: Fermi: -0.62 to 0.54 mL/min/g; Tikhonov: -0.83 to 0.63 mL/min/g; TSVD: -0.64 to 0.57 mL/min/g.

### Comparison with PET

Median interval between CMR and PET was 7 days (interquartile range 4–25). Resting heart rate (54 ± 6 v 55 ± 6 bpm; p = 0.71), systolic blood pressure (118 ± 14 v 125 ± 18 mmHg; p = 0.14) and rate pressure product (6493 ± 870 v 7108 ± 1258 mmHg.bpm; p = 0.18), as well as stress heart rate (75 ± 9 v 73 ± 13 bpm; p = 0.46), systolic blood pressure (110 ± 13 v 109 ± 13 mmHg; p = 0.66) and rate pressure product (8230 v 7947 mmHg.bpm; p = 0.46) did not differ significantly between CMR and PET scans. Quantitative PET analysis was not possible in one patient due to substantial movement artefact.

**Figure 3:**
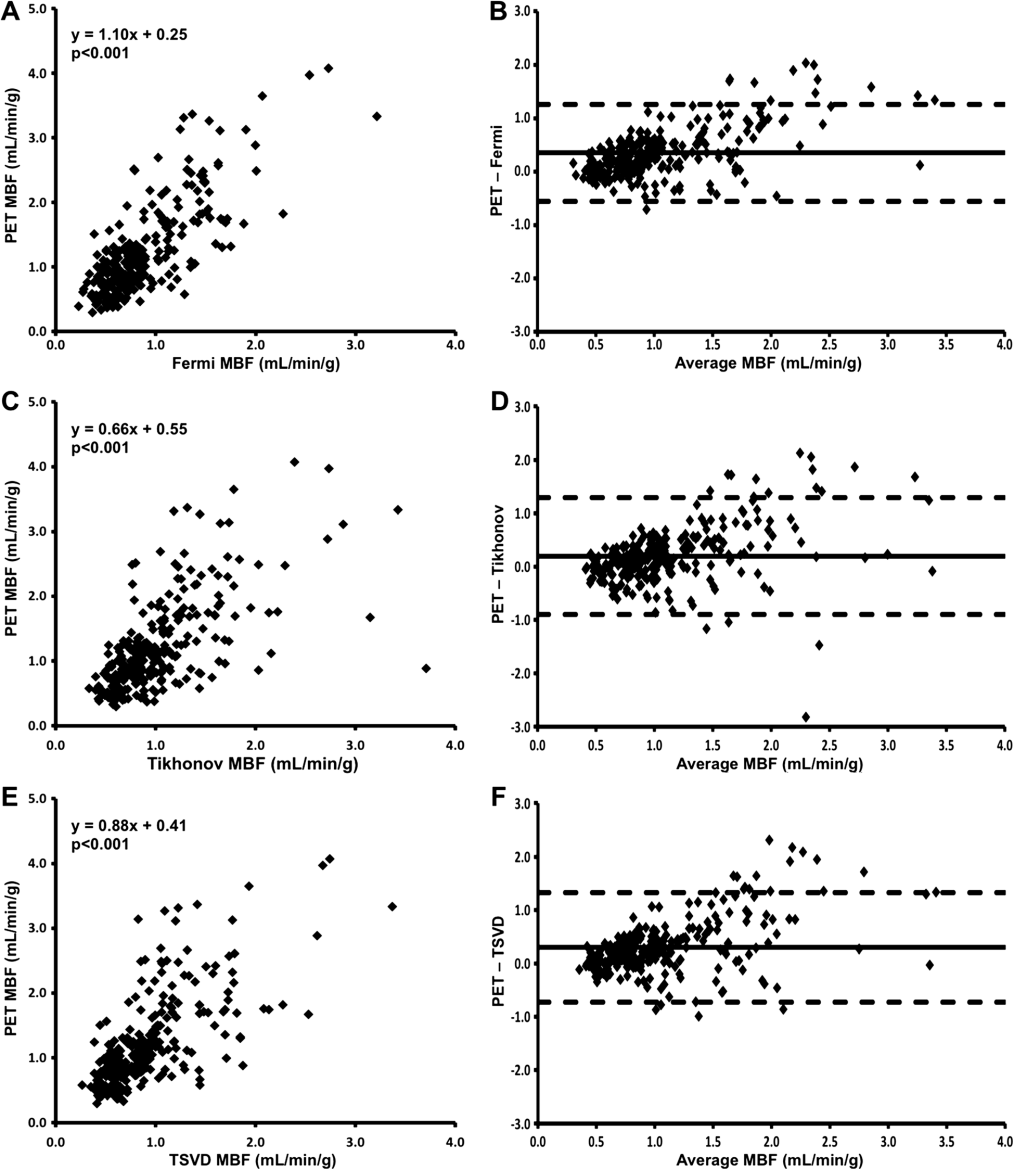
**Comparison of CMR and PET-derived MBF.** CMR-derived MBF measured using Fermi function parameterization **(Fermi, A)**, Tikhonov regularization **(Tikhonov, C)** and TSVD **(E)** deconvolution methods plotted against PET-derived MBF, with corresponding Bland-Altman plots (**B, D, F** respectively).

**Figure 4:**
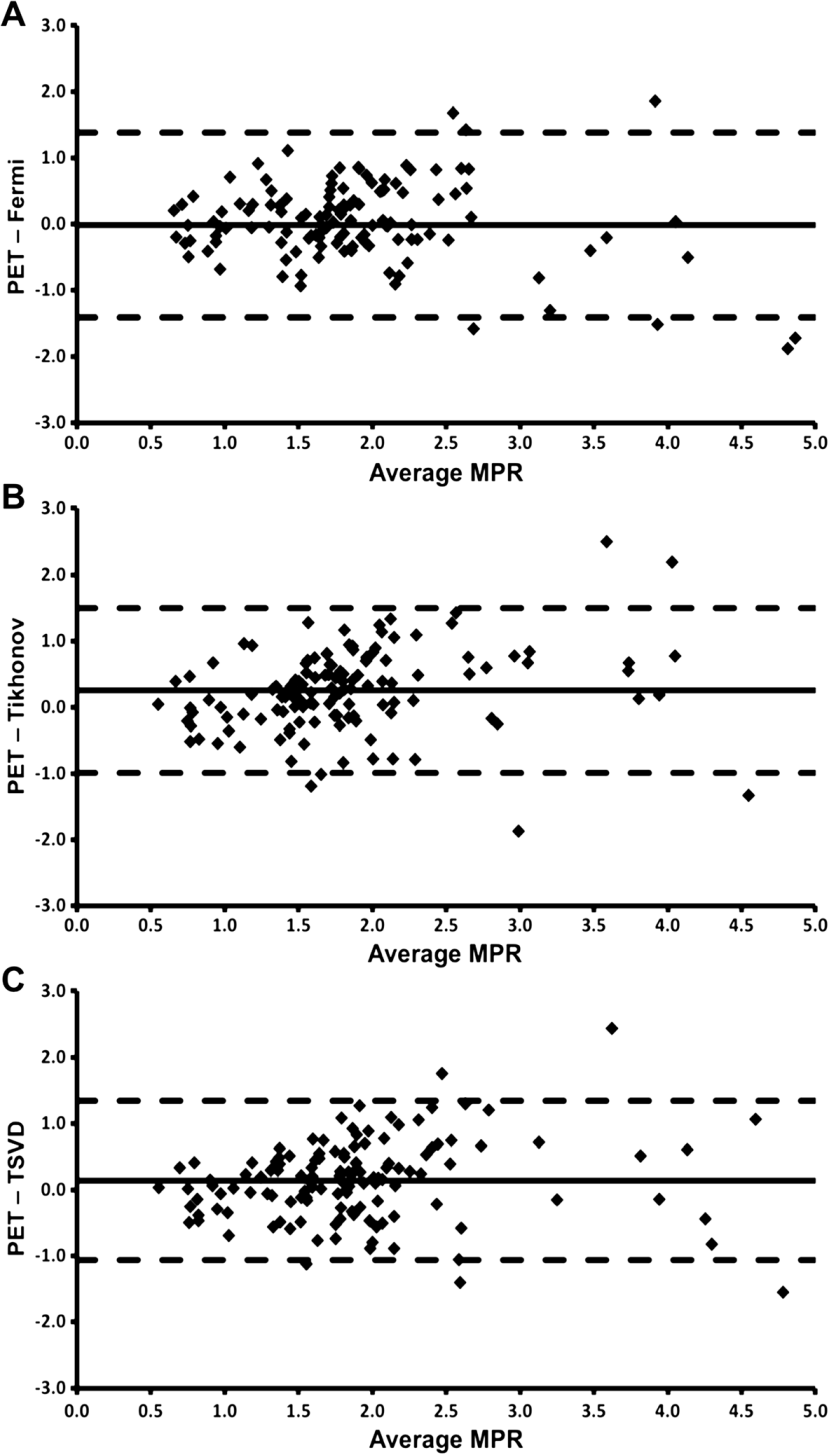
**Comparison of CMR and PET-derived MPR.** Bland-Altman plots displaying the agreement between CMR MPR measured using Fermi function (Fermi), Tikhonov regularization (Tikhonov) and TSVD deconvolution methods and MPR measured using PET.

There was a significant linear relationship between CMR-derived MBF and PET-derived MBF using each CMR deconvolution method (p < 0.001 for each using GEE; Figure 3). For each method of deconvolution, the within-subject and between-subject correlations between CMR-derived MBF and PET-derived MBF were significant (Fermi-CMR v PET: within-subject *r* = 0.63, p < 0.001; between-subject *r* = 0.91, p < 0.01; Tikhonov-CMR v PET: within-subject *r* = 0.47, p < 0.001; between-subject *r* = 0.81, p < 0.02; TSVD-CMR v PET: within-subject *r* = 0.53, p < 0.001; between-subject *r* = 0.82, p < 0.02). Nevertheless, as is evident from Table 4 and the Bland-Altman plots in Figure 3, mean CMR-derived MBF, using each of the deconvolution methods, was significantly lower than mean PET-derived MBF. The magnitude of the difference increased as blood flow increased, although there was greater heterogeneity at higher blood flow values. In spite of this however, mean CMR-derived MPR measured using Fermi and TSVD deconvolution methods was not significantly different from PET-derived MPR (Table 4, Figure 4). Whilst mean Tikhonov-CMR-derived MPR was significantly lower than PET-derived MPR, the absolute difference was small and the bias was consistent across the entire range of MPR values. Agreement between PET-derived MPR and MPR measured using each of the CMR deconvolution methods was moderate.

**Table 4:** Comparison of CMR and PET-derived myocardial blood flow and myocardial reserve index

**A. Mean values**				
	**PET**	**Fermi**	**Tikhonov**	**TSVD**
Overall MBF (mL/min/g)	1.23 ± 0.72	0.90 ± 0.44	1.05 ± 0.51	0.94 ± 0.47
Stress MBF (mL/min/g)	1.62 ± 0.81	1.16 ± 0.47	1.29 ± 0.51	1.21 ± 0.50
Rest MBF (mL/min/g)	0.84 ± 0.27	0.64 ± 0.20	0.80 ± 0.35	0.67 ± 0.17
MPR	1.97 ± 0.87	1.97 ± 1.13	1.73 ± 0.73	1.87 ± 0.79
**B. Mean differences and limits of agreement**				
	**Mean difference**	**p-value**	**95% limits of agreement**	
**Overall MBF (mL/min/g)**				
PET – Fermi	0.35	<0.001	−0.56 to 1.26	
PET – Tikhonov	0.19	0.014	−0.90 to 1.29	
PET – TSVD	0.30	<0.001	−0.73 to 1.32	
**Stress MBF (mL/min/g)**				
PET – Fermi	0.48	<0.001	−0.65 to 1.61	
PET – Tikhonov	0.34	0.004	−0.95 to 1.63	
PET – TSVD	0.43	0.001	−0.89 to 1.75	
**Rest MBF (mL/min/g)**				
PET – Fermi	0.22	<0.001	−0.28 to 0.72	
PET – Tikhonov	0.05	0.531	−0.72 to 0.82	
PET – TSVD	0.17	<0.001	−0.32 to 0.66	
**MPR**				
PET – Fermi	0.02	0.869	−1.41 to 1.38	
PET – Tikhonov	0.25	0.028	−0.99 to 1.50	
PET – TSVD	0.14	0.200	−1.06 to 1.34	

95% limits of agreement represent mean difference ± 2 standard deviations. Other abbreviations as per previous Tables.

**Figure 5:**
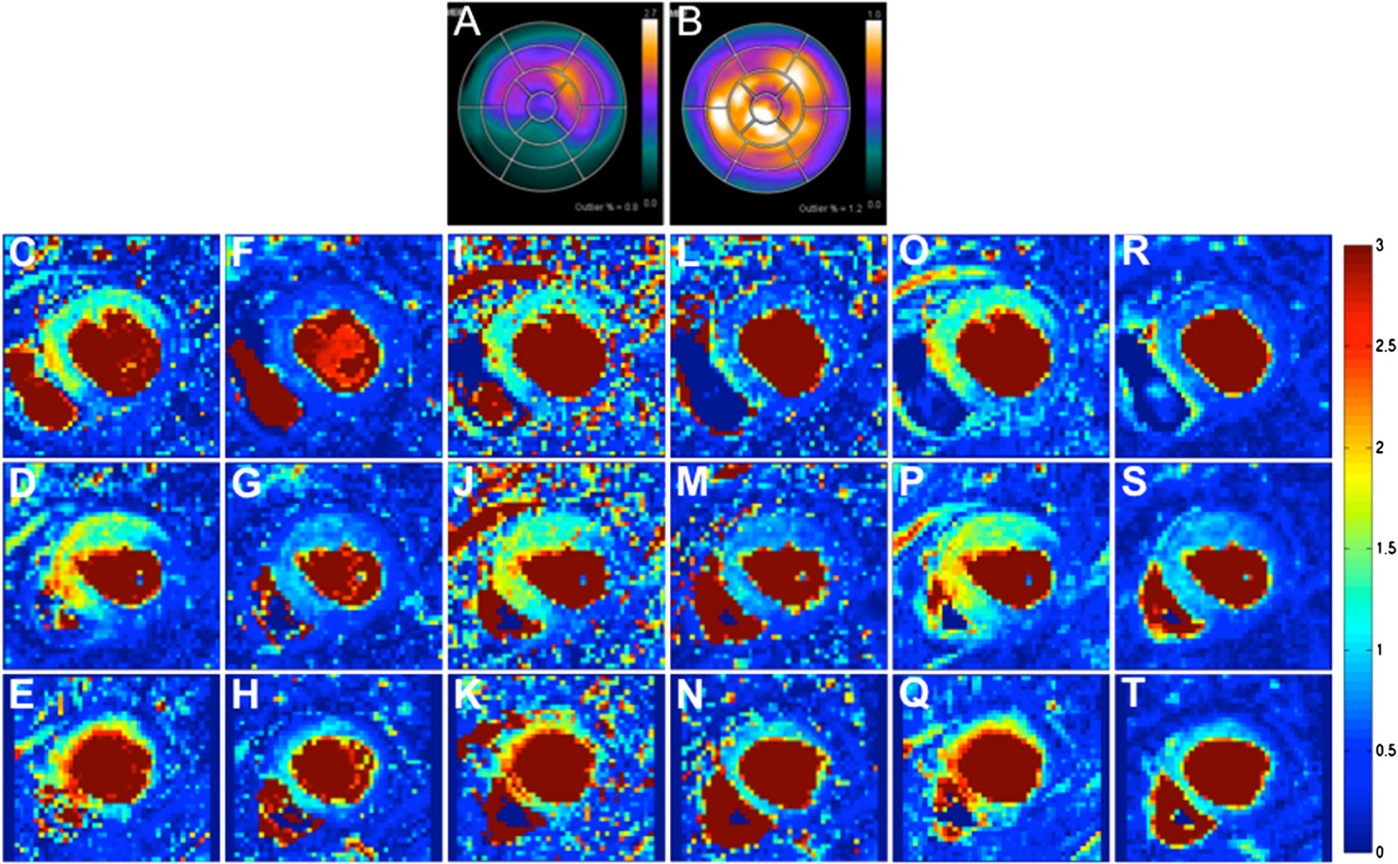
**Example voxel-wise MBF maps.** CMR MBF maps quantified using Fermi function parameterization (stress **C-E**, rest **F-H**; basal-ventricular **C** and **F**, mid-ventricular **D** and **G**, apical-ventricular **E** and **H**), Tikhonov regularization **(I-N)** and TSVD **(O-T)**, with corresponding stress **(A)** and rest **(B)** PET polar plots in a patient with a significant stenosis of the right coronary artery.

Significantly lower mean stress MBF and MPR values were seen in stenotic coronary territories compared to remote territories with PET and with each of the CMR deconvolution methods (Table 5, Figure 5). No difference was seen in mean resting MBF between stenotic and remote territories using CMR, although a small difference was seen with PET.

**Table 5:** CMR and PET-derived myocardial blood flow and myocardial reserve index in stenotic versus non-stenotic coronary territories

	**Stenotic territory**	**Remote territory**	**p value**
**Stress MBF (mL/min/g)**			
PET	1.20 ± 0.66	1.89 ± 0.78	<0.001
Fermi	0.81 ± 0.29	1.36 ± 0.47	<0.001
Tikhonov	0.99 ± 0.36	1.47 ± 0.55	<0.001
TSVD	0.90 ± 0.33	1.38 ± 0.54	<0.001
**Rest MBF (mL/min/g)**			
PET	0.80 ± 0.26	0.86 ± 0.28	0.034
Fermi	0.62 ± 0.18	0.62 ± 0.17	0.917
Tikhonov	0.79 ± 0.46	0.79 ± 0.24	0.926
TSVD	0.66 ± 0.20	0.68 ± 0.17	0.618
**MPR**			
PET	1.53 ± 0.68	2.25 ± 0.88	<0.001
Fermi	1.35 ± 0.47	2.40 ± 1.31	<0.001
Tikhonov	1.35 ± 0.47	1.95 ± 0.79	0.004
TSVD	1.41 ± 0.49	2.10 ± 0.86	<0.001

## Discussion

This paper demonstrates the differences in CMR-derived measurement of MBF that result from common variations in quantification methodology. Although we have used voxel-wise analysis, the methodological steps assessed are generic and would be expected to impact on segmental/sector-wise analysis similarly. In addition, this study represents the first validation of voxel-wise CMR MBF quantification against PET in patients with CAD.

CMR is an attractive alternative to PET for measurement of MBF. Advantages of CMR include higher spatial and temporal resolution, more accurate endocardial border definition hence less potential for blood pool spill-over, wider availability, absence of ionizing radiation and its multiparametric nature which allows blood flow to be interpreted in the context of accurate functional and viability data obtained during the same scan [26]. Disadvantages include vulnerability to arrhythmias, contrast agents that are potentially toxic in severe renal impairment and that have both intra- and extravascular components, susceptibility artefact and the need for prolonged respiratory suspension. Nevertheless, CMR MBF quantification has been increasingly applied in the research setting.

The study cohort was chosen in order to ensure substantial regional variation in MBF, thus allowing evaluation of methodological differences in CMR MBF quantification over a wide range of MBF values and meaningful segmental comparison between CMR and PET. Reflecting the extent of CAD (59 of 144 segments (41%) had visually apparent perfusion defects on qualitative CMR analysis), mean stress MBF and MPR measured with both CMR and PET in patients with CAD were relatively low.

### Saturation effects and AIF location

Despite using a contrast agent dose that is half of that most commonly used clinically, Figure 1A demonstrates that considerable saturation effects remained and highlights the importance of accounting for the non-linear relationship between contrast agent concentration and signal intensity and correcting for signal saturation. Higher MBF values were also observed when AIF(t) was extracted from the blood pool in the mid- compared to the basal-ventricular short-axis LV slice (Figure 1B). It is likely that inadvertent inclusion of trabeculae within the mid-ventricular AIF ROI, which were more difficult to avoid in the smaller mid-ventricular cavity, had a similar truncating effect, hence the use of basal images for AIF extraction throughout the remainder of the current study. Potential differences in contrast-blood mixing between ventricular levels may also have contributed to the observed difference in estimated MBF.

### Deconvolution methods

Many deconvolution algorithms have been described, which can broadly be classified into parametric and non-parametric techniques. Parametric deconvolution assumes a shape for the tissue impulse response function. Fermi function parameterization, the most widely applied parametric deconvolution method for MBF quantification, is relatively straightforward to apply and MBF measurements using this method have been validated against microspheres [7,27]. The main disadvantage of Fermi parameterization is that the assumed form of the impulse response function may not be appropriate, potentially leading to systematic errors in MBF estimation. Non-parametric approaches do not assume a functional form for the tissue impulse response function, however due to the numerically unstable nature of the deconvolution process, constraints in the form of regularization are still required. The solution obtained depends on the choice of regularization parameter. Tikhonov regularization, which results in a smooth truncation of the singular values, allows the use of methods such as L-curve analysis or generalized cross-validation to find the optimum regularization parameter directly from the data. This offers potential advantages over TSVD in which an arbitrary threshold (commonly set at 20% of the maximum singular value) is used. Tikhonov regularization, combined with an additional constraint of a b-spline representation of the impulse response function, is the most commonly applied non-parametric technique for estimating MBF and has been validated with microspheres [19,20]. However the implementation is considerably more involved than that of either Fermi parameterization or TSVD.

In this study, the difference in estimated MBF between deconvolution techniques was minimal. Indeed, the 95% limits of agreement for measurement of MBF between deconvolution techniques were similar (or smaller) in magnitude to the limits of agreement between AIF extraction locations (i.e. basal versus mid-ventricular slice) and to the limits of agreement between observers. In keeping with these findings Pack et al. [28], who compared four quantitative analysis techniques in healthy volunteers (although with non-identical doses of contrast agent and without external validation), found no difference in MBF measurements between a model-free deconvolution technique, a two-compartment model and Patlak plot analysis, although stress MBF was higher with Fermi function parameterization. In the current study, MBF measured using each deconvolution technique showed a significant correlation with PET-derived MBF, with correlation coefficients that are in keeping with previous studies comparing sector-wise CMR MBF measurements with PET [8,9]. Perhaps most importantly, there was a very clear demarcation in mean stress MBF and MPR between stenotic and remote coronary territories using each quantification method.

### Comparison with PET

MBF measured using Fermi parameterization showed the closest correlation with PET-derived MBF, whereas the non-parametric techniques, particular Tikhonov regularization, displayed greater heterogeneity. As a possible explanation for these findings, Zarinabad et al. [29] demonstrated voxel-wise MBF quantification with Fermi analysis to be most robust to noise in a physiologically realistic two-compartment myocardial perfusion phantom, whereas Tikhonov regularization was the least robust. Nevertheless, each of the CMR quantification techniques in the current study underestimated MBF compared to PET, the degree of which became more apparent as MBF increased. Whilst the dose of contrast agent and method of accounting for saturation used here are well described [11], that this effect was seen with all deconvolution methods could suggest an inaccuracy in the saturation correction algorithm. However Hsu et al. [10], who evaluated voxel-wise CMR MBF quantification against microspheres in a canine model with a dual-bolus technique, also found CMR (parametric deconvolution analysis) to underestimate MBF, the magnitude of which increased as MBF increased. The authors demonstrated that the underestimation was not due to an issue intrinsic to voxel-level quantification, with the same degree of underestimation seen with sector-wise analysis. Parkka et al. [8] and Fritz-Hansen et al. [30], who compared sector-wise CMR-derived MBF against O-15 and N-13 PET respectively in healthy volunteers, also found CMR to underestimate MBF compared to PET, again with greater underestimation at higher MBF values. In contrast Morton et al. [9], who compared sector-wise CMR MBF (dual-bolus technique, Fermi analysis) with N-15 PET in patients with CAD, found mean MBF to be higher with CMR than with PET although the significance of the difference was not stated. Nevertheless as is evident from their presented Bland-Altman plots, while CMR overestimated MBF at low MBF values, CMR underestimated MBF at higher MBF values. The reasons for these consistent differences between CMR and PET are not clear, but may relate to differences in quantification methodology between modalities.

In the current study, the underestimation of both rest and stress MBF was largely cancelled out by calculation of MPR, which was not significantly different from PET using Fermi and TSVD methods although a small underestimation was seen with Tikhonov regularization, findings which are consistent with the other discussed studies [8,9,30].

### Precision

Interobserver repeatability for MBF quantification with CMR has not been widely reported. 95% limits of agreement were used to assess interobserver repeatability here in order to allow comparison with variability due to the investigated methodological differences, but they have not previously been reported. Morton et al. [9], using Fermi function parameterization in patients with CAD, reported interobserver coefficients of variation of 16% and 18% respectively for stress and rest MBF using sector-wise (coronary territory) analysis with automated myocardial border detection. The slightly higher coefficients of variation in the current study (stress MBF 24%, rest 26% for Fermi function) are likely to reflect the segmental-wise (rather than per coronary territory) comparison between observers and the manual method of defining the myocardial borders (as well as the AIF ROI and right ventricular septal insertion point), both of which would inherently be associated with greater variability.

### Limitations

The number of patients with CAD included was small and reflects the reticence of such patients to undergo two additional investigations prior to PCI. Nevertheless, overall sample size was comparable to other methodological studies of this type and with appropriate statistical adjustment, segmental analysis allowed for meaningful investigation. The methodological steps evaluated are not exhaustive, but do represent some of the major variations in MBF quantification.

The selection of patients with CAD required >75% coronary luminal stenosis as determined visually. The limitations of visual assessment of angiography are well recognized however the purpose of recruiting such patients was simply to ensure substantial regional variation in MBF, which was achieved. (We did not aim to assess diagnostic performance). In any case, in all patients the degree of coronary disease was felt sufficient to warrant PCI clinically.

It is recognized that Rb-82 PET is not an ideal gold standard, with myocardial extraction of Rb-82 known to become non-linear as flow increases. Correction algorithms, which have generally been developed in small numbers of healthy volunteers only, are therefore required for MBF estimation and inevitably lead to inaccuracies [22]. Nevertheless Rb-82 PET has been well validated and does provide an external validation of the CMR-derived MBF measurements and as discussed, our findings are in keeping with most previous comparisons of sector-wise CMR MBF quantification with N-13 and O-15 PET [22,31].

## Conclusions

This paper demonstrates the feasibility of voxel-wise CMR quantification of MBF in patients with CAD and healthy volunteers and shows the effect that differences in quantification methodology have on MBF measurements. The magnitude of the difference in estimated MBF between deconvolution methods is no greater than differences due to simple methodological factors such as short-axis slice used for AIF extraction, or indeed differences between observers. Standardization of the quantification process will aid comparison between studies and may help CMR MBF quantification enter clinical use.

## Abbreviations

AIF: Arterial input function; CAD: Coronary artery disease; CMR: Cardiovascular magnetic resonance; CT: Computed tomography; EF: Ejection fraction; LV: Left ventricle; MBF: Myocardial blood flow; MPR: Myocardial perfusion reserve; PET: Positron emission tomography; TSVD: Truncated singular valued decomposition.

## Competing interests

The authors declare that they have no competing interests.

## Authors’ contributions

All authors have contributed significantly to the work. CAM, JHN and MS conceived and directed the project. JHN and CAM developed the CMR perfusion analysis tools, with input from AB, PW, CDS and GPM. CAM, MPA and RME recruited the patients. DC, CAM, MPA and MS performed the CMR scanning and analysis. CT, DT and PA performed the PET scanning and analysis. All authors provided critical review of the manuscript. All authors read and approved the final manuscript.
